# Myeloid cell-specific deletion of *Capns1* prevents macrophage polarization toward the M1 phenotype and reduces interstitial lung disease in the bleomycin model of systemic sclerosis

**DOI:** 10.1186/s13075-022-02833-7

**Published:** 2022-06-21

**Authors:** Li Zhang, Dong Zheng, Yuemei Yan, Yong Yu, Ruizhen Chen, Zheng Li, Peter A. Greer, Tianqing Peng, Qiang Wang

**Affiliations:** 1 Department of Dermatology, Zhongshan Hospital, Fudan University, Xuhui District, 180 Fenglin Road, Shanghai, 200032 People’s Republic of China; 2 Department of Dermatology, Huashan Hospital, Fudan University, 12 Wulumuqi Middle Road, Shanghai, 200040 People’s Republic of China; 3 Centre of Clinical Laboratory, The First Affiliated Hospital of Soochow University, Suzhou, Jiangsu 215006 People’s Republic of China; 4 Key Laboratory of Viral Heart Diseases, Ministry of Public Health, Shanghai Institute of Cardiovascular Diseases, Zhongshan Hospital, Fudan University, 180 Fenglin Road, Shanghai, 200032 People’s Republic of China; 5 Center of Laboratory, Zhongshan Hospital, Fudan University, 180 Fenglin Road, Shanghai, 200032 People’s Republic of China; 6 Department of Pathology and Molecular Medicine, Division of Cancer Biology and Genetics, Queen’s University, Queen’s Cancer Research Institute, Kingston, ON Canada; 7 Departments of Medicine, Pathology and Laboratory Medicine, Lawson Health Research Institute of London Health Sciences Centre, Western University, 800 Commissioners Road, London, ON N6A 5W9 Canada

**Keywords:** Calpains, Lung, Macrophage, Polarization, Systemic sclerosis, Bleomycin model

## Abstract

**Background:**

Calpains are a family of calcium-dependent thiol proteases that participate in a wide variety of biological activities. In our recent study, calpain is increased in the sera of scleroderma or systemic sclerosis (SSc). However, the role of calpain in interstitial lung disease (ILD) has not been reported. ILD is a severe complication of SSc, which is the leading cause of death in SSc. The pathogenesis of SSc-related ILD remains incompletely understood. This study investigated the role of myeloid cell calpain in SSc-related ILD.

**Methods:**

A novel line of mice with myeloid cell-specific deletion of *Capns1* (*Capns1-ko*) was created. SSc-related ILD was induced in *Capns1-ko* mice and their wild-type littermates by injection 0.l mL of bleomycin (0.4 mg/mL) for 4 weeks. In a separate experiment, a pharmacological inhibitor of calpain PD150606 (Biomol, USA, 3 mg/kg/day, i.p.) daily for 30 days was given to mice after bleomycin injection on daily basis. At the end of the experiment, the animals were killed, skin and lung tissues were collected for the following analysis. Inflammation, fibrosis and calpain activity and cytokines were assessed by histological examinations and ELISA, and immunohistochemical analyses, western blot analysis and Flow cytometry analysis.

**Results:**

Calpain activities increased in SSc-mouse lungs. Both deletion of *Capns1* and administration of PD150606 attenuated dermal sclerosis as evidenced by a reduction of skin thickness and reduced interstitial fibrosis and inflammation in bleomycin model of SSc mice. These effects of reduced calpain expression or activity were associated with prevention of macrophage polarization toward M1 phenotype and consequent reduced production of pro-inflammatory cytokines including TNF-α, IL-12 and IL-23 in lung tissues of *Capns1-ko* mice with bleomycin model of SSc. Furthermore, inhibition of calpain correlated with an increase in the protein levels of PI3K and phosphorylated AKT1 in lung tissues of the bleomycin model of SSc mice.

**Conclusions:**

This study for the first time demonstrates that the role of myeloid cell calpain may be promotion of macrophage M1 polarization and pro-inflammatory responses related PI3K/AKT1 signaling. Thus, myeloid cell calpain may be a potential therapeutic target for bleomycin model of SSc-related ILD.

## Introduction

Scleroderma or systemic sclerosis (SSc) is an autoimmune disease characterized by vasculopathy, immune system activation and fibrosis of the skin [1], accompanied by various complications in internal organs. Studies have revealed that approximately 80% patients with SSc display lung fibrosis and 25% to 30% patients develop progressive interstitial lung disease (ILD) [2], which is the leading cause of morbidity and mortality in patients with SSc [3, 4]. However, the mechanisms underlying SSc-related ILD remain unclear and effective therapies are limited.

Calpains are a family of calcium-dependent thiol proteases that participate in a wide variety of biological activities [5, 6]. The two most extensively studied isoforms, calpain-1 and calpain-2, are heterodimers, differing in their calcium requirement for activation (~ 50 μM for calpain-1 and ~ 1000 μM for calpain-2) [7]. They consist of a distinct large 80-kDa catalytic subunit encoded by the genes *Capn1* and *Capn2*, respectively, and a common small 28-kDa regulatory subunit encoded by *Capns1*. The small subunit is indispensable for calpain-1 and calpain-2 stability and activity. Thus, *Capns1* knock-out induces the impairment of calpain-1 and calpain-2 activity [8]. Calpain has been implicated in lung fibrosis [9–11]. For example, calpain mediates EGF- and PDGF-induced collagen synthesis and proliferation of pulmonary artery smooth muscle cells via an intracrine TGF-β1 pathway in pulmonary hypertension [12]. However, it has never been reported whether calpain plays a role in development of SSc-related ILD.

Macrophages are critical effectors and regulators of inflammation and the innate immune response, the immediate, pre-programmed arm of the immune system [13]. Human alveolar macrophages (AMs) express high levels of interleukin (IL)-10, IL-13, and platelet derived growth factor (PDGF) in patients with idiopathic pulmonary fibrosis (IPF) and SSc [14–18]. It has also been shown that alveolar macrophages produce elevated levels of chemokines and increased expression of CD206 (macrophage mannose receptor 1), an alternatively activated macrophage marker, in several fibrotic lung diseases including SSc-related ILD [19]. These previous findings underscore an important role of alveolar macrophage activation in SSc-related ILD. However, the mechanisms underlying macrophage activation remain not fully understood in SSc-related ILD. It is well known that macrophage polarization toward M1 phenotype is important to promote inflammation. Our recent study demonstrated that calpain-2 facilitated macrophage M1 polarizations through NF-κB and STAT3 signaling pathways [20], suggesting a role of calpain in macrophage polarization. Nevertheless, it remains to be determined how calpain mediates macrophage polarization toward M1 phenotype. A previous study showed that Akt1 and Akt2 played central but opposite roles in the control of macrophage responsiveness and inflammation [21]

In this study, we hypothesized that calpain activation promotes macrophage polarization toward M1 phenotype by relating the PI3K/AKT signaling, leading to ILD in bleomycin model of SSc, and that myeloid cell-specific *Capns1-ko* prevents macrophage polarization toward M1 phenotype thereby reducing bleomycin model of SSc-related ILD in mice.

## Materials and methods

### Animals

All animal procedures were approved by the Institutional Animal Care and Use committee of Zhongshan Hospital Fudan University (SYXK2016-0006) in compliance with the guidelines for the Care and Use of Laboratory Animals published by the National Academy Press (NIH Publication No. 85–23, revised 1996). Mice with myeloid cell-specific deletion of *Capns1* (*Capns1*-ko) were generated by breeding mice bearing the targeted *Capns1*^*PZ*^ allele containing *loxP* sites flanking essential coding exons and mice with myeloid cell-specific expression of Cre recombinase under the control of myeloid cell-specific LYZ promoter as we recently described [22, 23]. The targeted *Capns1*^PZ^ allele and transgenic Cre were identified by PCR as previously described [22, 23]. All mice used in this study including controls were littermates of the same generation. *Capns1*^*PZ/PZ*^ mice were used as wild-type control for *Capns1-ko* groups.

### Experimental protocols

SSc was induced by subcutaneous injection of bleomycin (BLM) (Nippon Kayaku Co. Ltd, Tokyo, Japan) as previously described [24]. Bleomycin (BLM) at a concentration of 0.4 mg/ml was injected intracutaneusly into the backs of model mice 0.1 ml/day daily for 28 days, while the control mice were given solution of PBS at the same dose. Briefly, female mice (age of 6 weeks) were allocated into four experimental groups (10 mice in each group): (A) Wild-type sham group: received 0.1 mL of PBS; (B) Wild-type BLM group: received 0.l mL of BLM (0.4 mg/mL); (C) *Capns1-ko* sham group: received 0.1 mL of PBS; and (D) *Capns1-ko* BLM group: received 0.1 mL of BLM (0.4 mg/mL). Thirty days after BLM injection, mice were subjected to the following experiments.

In a separate experiment, 24 h after BLM injection mice (C3H/He, female, 6-week old) were treated with 0.1 mL of PD150606 (Biomol, USA, 3 mg/kg/day, i.p.) daily for 30 days [25]. PD150606 was separately dissolved in DMSO (concentration < 0.1%) and further diluted in PBS. Four experimental groups (10 mice in each group) were included: (A) Sham group: received PBS; (B) BLM group: received BLM; (C) PBS + BLM group: received BLM + PBS; and (D) PD150606 + BLM group: received BLM + PD150606. The animals were sacrificed 30 days after BLM injection. The animal use protocol was approved by the Institutional Animal Care and Use Committee at the Zhongshan Hospital, Fudan University(ethics approval number: 2016–130).

### Reagents

Rabbit anti-rat calpain1 polyclonal antibody (Abcam-28258, USA), rabbit anti-rat calpain2 polyclonal antibody (Abcam-236650, USA), rabbit anti-rat CD8 antibody (Santa Cruz Biotechnology-53212), rabbit anti-rat CD4 antibody (Abcam-207755, USA), rabbit anti-rat CD68 antibody (Abcam-125212, USA), rabbit anti-mouse Tumor necrosis factor-alpha (TNF-α) antibody (Santa Cruz Biotechnology-130349), rat anti-mouse F4/80 (BM8) monoclonal antibody (Santa Cruz Biotechnology-52664), mouse anti-human IL-10 (E-10) monoclonal antibody (Santa Cruz Biotechnology-8438), mouse anti-human IL-12 p70 (14L7) monoclonal antibody (Santa Cruz Biotechnology-74150), mouse anti-human IL-23 (D-12) monoclonal antibody (Santa Cruz Biotechnology-271349), COL3A1 (B-10) monoclonal antibody (Santa Cruz Biotechnology-271249), mouse anti-human COL1A1 (3G3) monoclonal antibody (Santa Cruz Biotechnology-293182), Rabbit anti-rat transforming growth factor-beta1 (TGF-β1(V)) polyclonal antibody (Santa Cruz Biotechnology-130348), Rabbit anti-rat MHC Class II polyclonal antibody (Huabio, ER 1913–01), Rabbit anti-rat fibroblast activation protein-α (FAP) polyclonal antibody (ABclonal, Catalog No.: A6349), α-SMA(Cell signaling technology-19245), CD206(Cell signaling technology-24595) were purchased for immunohistochemistry (IHC) and western blot (WB). DAB kit(DAB-2031/2032) was purchased from Maxim Biological Company (http://www.maxim.com.cn/sitecn/myzhptsj/1074.html, Fuzhou, China), HRP-conjugated Monoclonal Mouse Anti-glyceraldehyde-3-phosphate Dehydrogenase (GAPDH) from KangChen Bio-tech (http://www.aksomics.com/index.php?c=article&id=1160,Shanghai, China), Trizol reagent from Invitrogen (USA), BCA reagent from TIANGEN BIOTECH (Beijing, China), and PD150606 from Biomol, USA. BLM from Nippon Kayaku Co. Ltd, Tokyo, Japan.

### Reverse transcription-polymerase chain reaction (RT-PCR)

RT-PCR was performed to analyze mRNA expression of *Capns1* and *Gapdh* as described in our recent study [26]. Shortly, tail biopsies of 3 mm from F1 mice were put in solution A(25 mM NaOH, 0.2 mM EDTA) 50 μl,95℃ for 45 min,cooling Room Temp for 130 min, then add solution B(40 mM Tris–HCL, PH 5.0) 50 μl to each tube, vortex 5 s, centrifuge 13000 rpm or 10000 g for 5 min. Each subsequent PCR (25 μl) contained 2 X PCR master 12.5 μl, Primer(10 μM) 0.4 μl, H2O 10.8 μl, mix 23 μl to each tube, add 1.3 μl DNA to each tube. PCR cycling was as follows: step 1, 3 min at 95 °C; 30 s at 95 °C, 40 s at 60 °C, and 40 s at 72 °C; and step 2, 31times 72℃ for 3 min, 4℃ forever. Cre Primers were as follows (5′ > 3′): Jax-Cre-F: 5'-GCGGTCTGGCAGTAAAAACTATC and Jax-Cre-R: 5'-GTGAAACAGCATTGCTGTCACTT. The PCR products were separated on 1.5% agarose gels, stained with ethidium bromide, and photographed under ultraviolet lights.

### Hematoxylin and eosin staining

For histological examination, 4-μm thick formalin-fixed and paraffin-embedded sections of skin tissues and lung tissues were stained with hematoxylin and eosin (H&E). Sections were evaluated by microscopy. Two independent observers randomly selected 5 fields of view (X200) for each slide. Extension of inflammation of lung tissue was graded into the following four categories [27]: grade 0, normal lung tissue without inflammation, scored 0; grade 1,minimal alveolitis ( +), widened alveolar septa due to inflammatory cells infiltration, the lesions confined to less than 20% of the whole lung, scored 1.0; grade 2, moderate alveolitis (+ +), the lesions extended to 20% to 50% of the whole lung, scored 2.0; grade 3, severe alveolitis (+ + +), diffuse lesions in more than 50% of the whole lung, scored 3.0. Also, extension of inflammation of skin tissue was graded into the following five categories[28]: Skin specimens were assessed and scored to provide a semiquantitative measurement of dermal inflammation (0, none; 1, little; 2, mild; 3, moderate; 4, severe).

### Masson's trichrome staining

For fibrotic analysis, 4-μm thick formalin-fixed and paraffin-embedded sections of skin tissues and lung tissues were stained with Masson trichrome staining according to standard techniques. Sections were evaluated by microscopy.

The images were analyzed using a Leica Qwin V3 System. Five fields of view (X200) were analyzed randomly for each slice with the collagen fiber staining blue as positive. Firstly all tissues were selected in the field of view (except the blank part) to record the total area of the tissue, then the blue part was selected to record the collagen area, and the collagen area Percentage (% collagen area = collagen area/total tissue area) was calculated. Image J was used to calculate the collagen volume fraction (CVF), which is the percentage of the blue area of collagen positive to the total area of the tissue.

### Hydroxyproline measurement

One hundred milligrams of lung and skin tissues were used to determine the content of hydroxyproline using the A030-2 hydroxyproline test kit (Nanjing JianCheng Bioengineering Institute (http://www.njjcbio.com/products.asp?id=337), Nanjing, China) following the manufacturer’s instructions. Hydroxyproline reflects the collagen metabolism of connective tissue diseases. Under the action of an oxidant, the generated oxidation product reacts with dimethylaminobenzaldehyde to show a purple-red color, and its content can be estimated according to the depth of its color. Hydroxyproline content (μg/mg wet weight) was measured as follows: (tested OD value – blank OD value)/(standard OD value – blank OD value) × standard sample concentration × total hydrolysate volume/tissue wet weight. The absorbance was read at 550 nm using a Diode Array spectrophotometer.

### Immunohistochemical analyses

Four-μm-thick sections were made. Sections were initially deparaffinized by xylene and dehydrated with ethanol. Endogenous peroxidase activity was blocked by 3% hydrogen peroxide in methanol at room temperature for 15 min, then dipped into ethylenediamine tetra-acetic acid (EDTA) to restore antigen. After cooling to room temperature, sections were incubated with the diluted primary antibodies calpain1 antibody(1:100), calpain2 antibody(1:100), TNF-α antibody(1:150), TGF-β1(V) antibody(1:100), CD4 antibody(1:100), CD8 antibody(1:100),F4/8 antibody (1:100),CD206 antibody(1:200), α-SMA antibody(1:200), FAP antibody(1:200) and MHC-II (1:400) in a wet box at 4 °C overnight. The next day, sections were incubated with secondary antibody and EnVision (ChemMafeTM EnVision^+^/HRP). The reaction was then visualized with a 3,3’-diaminobenzidine (DAB) kit. Sections were counterstained with hematoxylin, dehydrated, and evaluated under light microscopy (Nikon, Japan). Lung tissue cells containing yellow granulation in the endochylema or nucleus were considered as positive. The number of positive cells was counted with Q500IW image analysis system (Leica, Germany) and Image-Pro Plus 6.0 software.

### Flow cytometry analysis

The ratio of M1/M2 macrophage cells in alveolar lavage fluid of mice was quantitated by flow cytometry (F4/80, CD206 and MHC II) according to the manufacturer’s instructions. Initially, 5 × 10^5^ cells were incubated with the following monoclonal antibodies: F4/80 (eBioscience, 11–4801-81), CD206 (BioLegend, 141,705)and MHC II (eBioscience,17–5321-81). Isotype matched controls were included. Cells were surface stained with antibodies on ice for 30 min, protected from light, and compared with unstained controls. Fluorescence-activated cell sorting (FACS) analysis was performed on a FACS Canto flow cytometer and analyzed using FACS Diva software (both from BD Bioscience). The lymphocyte population was selected, followed by gating on F4/80 cells, and then set to analyze the percentage of F4/80^+^CD206^+^ cells and F4/80^+^MHC II^+^ cells.

### Western blot analysis

Lung tissues were homogenized in a lysis buffer. Protein concentration was determined using a BCA kit. Twenty micrograms of proteins from each sample were subjected to electrophoresis on 12% sodium dodecyl sulfate–polyacrylamide gel (SDS-PAGE), and then transferred to polyvinylidene fluoride (PVDF) membranes on a semidry electro transferring unit (Bio-Rad, USA). PVDF membranes were blocked with 3% Albumin Bovine V for 2 h and incubated with the diluted primary antibodies against GAPDH(1:4000), calpain1(1:100), calpain2(1:100), pAkt1(1:100), pAkt2(1:100), Akt1(1:100), Akt2(1:100) and PI3K(1:100) overnight at 4 °C. After the overnight incubation with the primary antibody, membranes were washed by PBST (Phosphate Buffered Saline with 0.1% Tween-20) and incubated with HRP-conjugated secondary antibody for 2 h. After extensive washing, blots were detected with enhanced chemiluminescent autoradiography reagent (TIANGEN BIOTECH, China) according to the manufacturer's instruction. GAPDH (37 kD), calpain1 (80 kDa), calpain2 (80 kDa), pAkt1 (60 kDa), pAkt2 (60 kDa), Akt1 (60 kDa), Akt2 (60 kDa) and PI3K (110 kDa) were detected at the indicated approximate molecular sizes. The signal intensity was quantitatively analyzed with Quantity-One analysis software.

### Determination of calpain activities

Ten micrograms of lung tissues were homogenized on ice. The tissue lysates were assayed for calpain activities using a commercially available kit (Catalog #K240-100,BioVision Co.). According to the manufacture’s instruction, obtain tissue’s supernatant to detect the protein concentration and adjust the protein concentration of each sample to be consistent. Then, add 5 μl of Calpain Substrate suc-LLVY-AMC, which can be cleaved by calpain, to each assay. Incubate at 37° C for 1 h in the dark and use a fluorescence counter to detect calpain activity. The fluorometric assay is based on the detection of cleavage of calpain substrate Ac-LLY-AFC.

### Statistical analysis

Data were means ± SD (standard deviation), unless otherwise indicated. The difference among multiple groups was determined by one-way analysis of variance (ANOVA) followed by Bonferroni. T-test was used for comparison between two groups. *P* values < 0.05 were considered statistically significant.

## Results

### Calpain activities are increased in bleomycin model of SSc-ILD mouse lung tissues

**Fig. 1:**
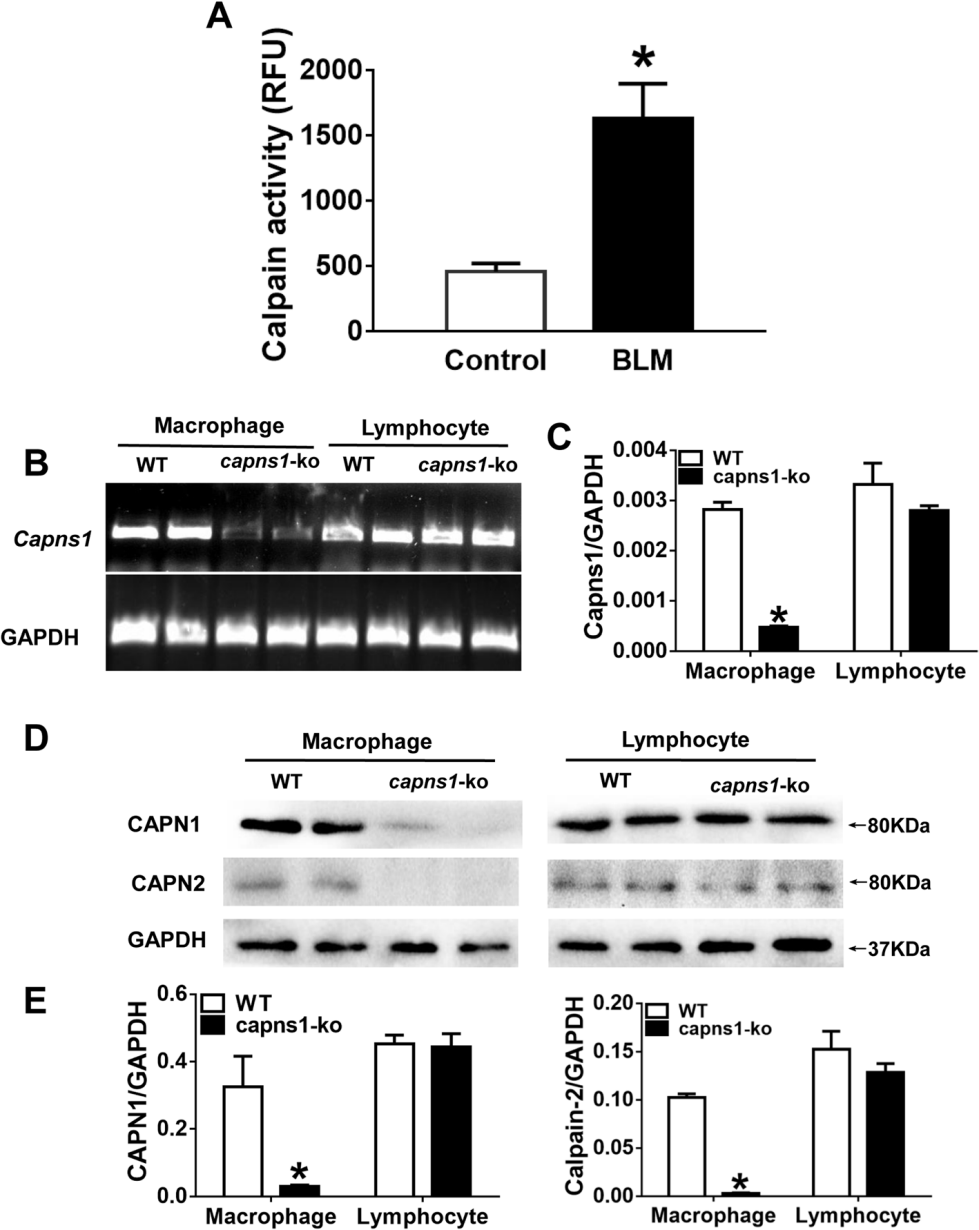
Calpain activity increases in bleomycin model of SSc-ILD and Characterization of mice with LYZ-specific *Capns1* knockout. **A** Calpain activity was measured in lysates of lung tissue from sham treated mice (Control) or mice treated with BLM. Data was compared by Student t-test. **B** Representative RT-PCR for GAPDH and *Capns1* mRNA from six mice in each group. Levels of *Capns1* mRNA were significantly reduced in *Capns1*-ko macrophages. In contrast, the *Capns1*-ko mRNA was not decreased in *Capns1*-ko lymphocytes. **C** *Capns1*-ko mRNA was detected by RT-PCR in wild-type and *Capns1*-ko macrophages and lymphocytes and normalized with GAPDH mRNA. The test of homogeneity of variance showed *P* > 0.1, ANOVA test was applied. **D** Representative western blots for CAPN1 and CAPN2 proteins from macrophages (left) and lymphocytes (right). **E** Both CAPN1 and CAPN2 protein levels were significantly reduced in *Capns1*-ko macrophage but not in lymphocytes. The test of homogeneity of variance showed *P* < 0.1, nonparametric test was applied. Data are mean ± SD. *n* = 6–10; *: *P* < 0.05, vs. sham group in WT mice; #: *P* < 0.05, vs. bleomycin model of SSc group in WT mice. *OD* optical density

Subcutaneous injection of bleomycin (BLM) in mice is an established model of SSc [24]. To establish calpain as a protease of interest in understanding the mechanisms behind ILD in SSc, we injected mice with BLM and measured calpain activity in lung tissues. As shown in Fig. 1, mouse lungs exhibited a significant increase in calpain activities after BLM treatment as compared to lungs of sham treated mice (1629.65 ± 647.15 vs. 456.66 ± 147.90, *p* = 0.006, Fig. 1A). This result demonstrates that calpain activity is induced in the lungs of mice by BLM treatment under conditions that model SSc/ILD.

### Characterization of myeloid cell-specific Capns1 knockout mice

To explore the role of calpain in myeloid cells in bleomycin model of SSc, we crossed *Capns1* floxed mice with LYZ-Cre transgenic mice to produced mice which were deficient for both calpain-1 and calpain-2 in the myeloid *Capns1-ko* lineages. To confirm that *Capns1* knockout was restricted to myeloid cells, *Capns1* mRNA was analyzed in macrophages and lymphocytes from *Capns1-ko* mice and their wild-type littermates (Fig. 1B). The mRNA levels of *Capns1* were significantly reduced in macrophages isolated from *Capns1-ko* mice compared with their wild-type littermates (*p* < 0.001, Fig. 1C). In contrast, *Capns1* mRNA levels in lymphocytes were similar between *Capns1-ko* mice and their wild-type littermates (*p* = 0.317, Fig. 1C). These results support myeloid cell-specific *Capns1* knockout using the LYZ-Cre transgene crossed with floxed *Capns1* mice.

Because the *Capns1*-encoded regulatory subunit is required for the stability as well as the proteolytic activities of calpain-1 and calpain-2 catalytic subunits (CAPN1 and CAPN2, respectively), we also analyzed the protein levels of CAPN1 and CAPN2 in macrophages (Fig. 1D). Both CAPN1 and CAPN2 protein levels were significantly reduced in macrophages isolated from *Capns1-ko* mice compared with their wild-type littermates (*p* = 0.047 and *p* < 0.001, respectively, Fig. 1E), confirming disruption of calpain-1 and calpain-2 in *Capns1-ko* mouse macrophages. Similar to the *Capns1* mRNA expression results, both CAPN1 and CAPN2 protein levels were not changed in lymphocytes between *Capns1-ko* mice and their wild-type littermates (*p* = 0.856 and *p* = 0.297, respectively, Fig. 1E). These results indicate successful knockout of *Capns1* in macrophages in *Capns1-ko* mice. These *Capns1-ko* mice displayed no overt phenotypes and were fertile.

### Myeloid deletion of Capns1 reduces dermal sclerosis in mouse of the bleomycin model of SSc

**Fig. 2:**
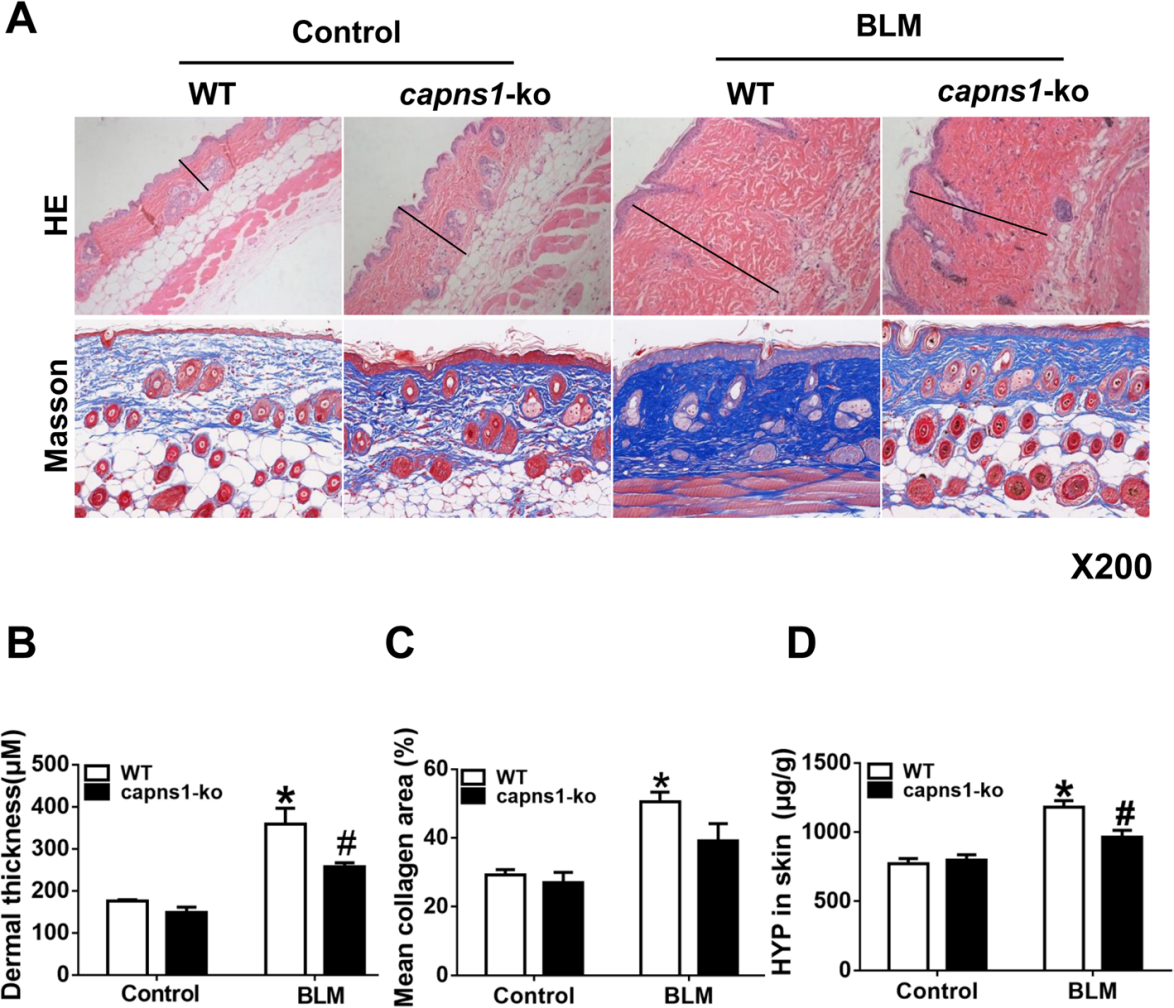
Histological analyses of inflammation and fibrosis in SSc skin tissues. **A** Skin sections of sham treated (Control) or BLM treated (bleomycin model of SSc) WT or Capns1-ko mice were stained with hematoxylin and eosin (H&E, upper, Scale bars, 50 μm, magnification X200) or Masson’s trichrome (lower, Scale bars, 100 μm, magnification X200). **B** Dermal thickness (µM) was quantified by histopathologic analysis of H&E stained sections. The test of homogeneity of variance showed *P* > 0.1, ANOVA test was applied. **C** Collagen deposition was quantified as the % Masson’s trichrome stained mean area. The test of homogeneity of variance showed P < 0.1, nonparametric test was applied. **D** The hydroxyproline (HYP) content in skin tissues was determined biochemically and expressed as µg HYP per gram of wet tissue. The test of homogeneity of variance showed *P* > 0.1, ANOVA test was applied. Data are mean ± SD. *n* = 8, *: *P* < 0.05, vs. Control WT mice; #: *P* < 0.05, vs. bleomycin model of SSc WT mice. *OD* optical density

Subcutaneous injection of BLM induced marked dermal sclerosis in mice as evidenced by increased dermal thickness, thickened and homogenous collagen bundles, thickening of vascular walls and inflammatory infiltrates (Fig. 2A, and *p* = 0.008, Fig. 2B). BLM also increased fibrosis as assessed either by Masson trichrome staining of collagen (Fig. 2A, and *p* = 0.003, Fig. 2C) or biochemical quantitation of hydroxyproline (HYP) as a surrogate measure of collagen (*p* = 0.003, Fig. 2D) in mouse skins. Histopathological examination revealed definite dermal sclerosis characterized by deposition of homogenous materials in the thickened dermis with cellular infiltrates in SSc WT mice relative to control WT mice. Masson’s trichrome staining showed a dense deposition of collagen in the thickened dermis in SSc WT mice. These effects of BLM were significantly attenuated in *Capns1-ko* mice, suggesting that myeloid cell-specific disruption of calpain-1 and calpain-2 reduces dermal sclerosis in mice.

### Myeloid deletion of Capns1 reduces ILD in a mouse of the bleomycin model of SSc

**Fig. 3:**
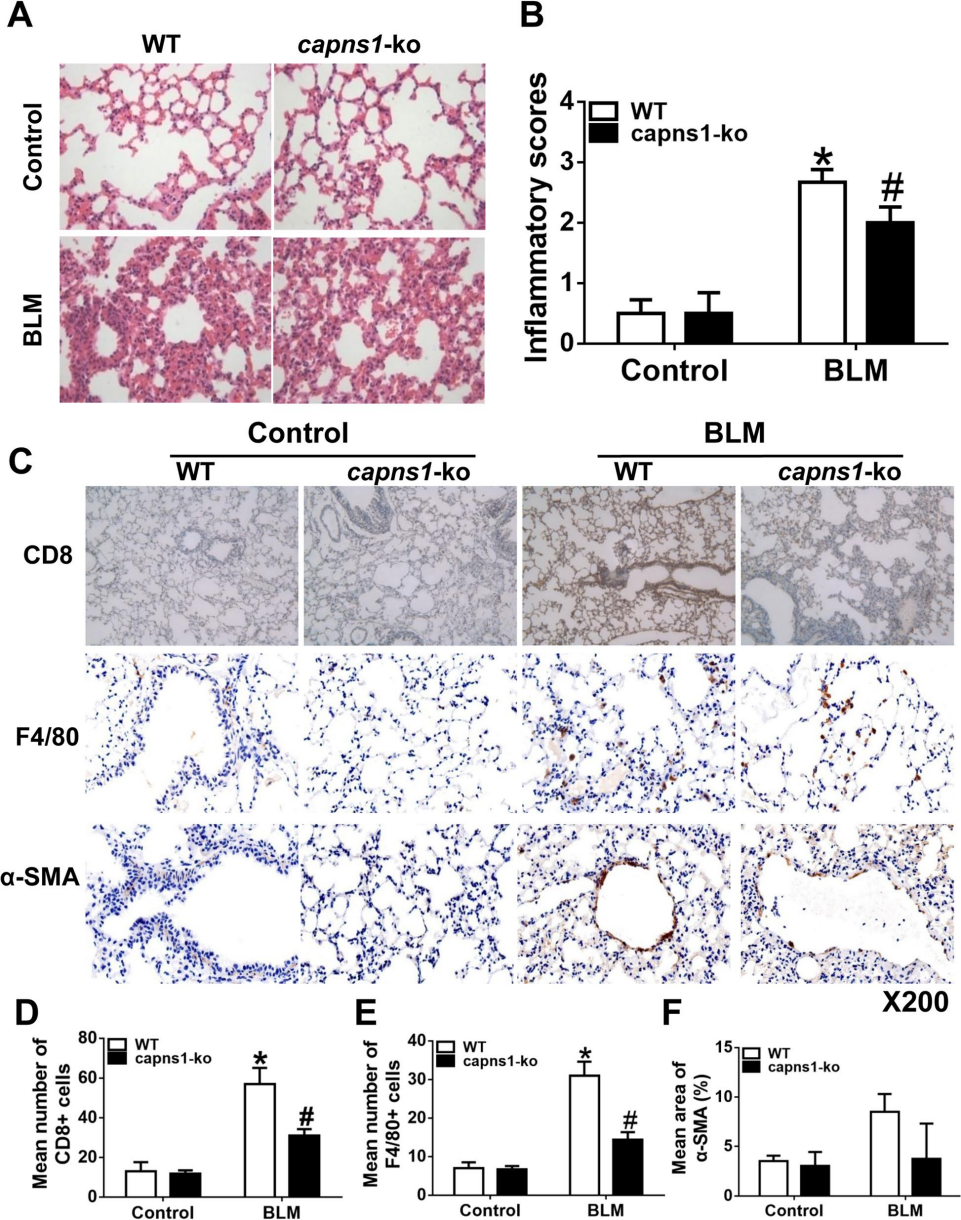
Analyses of lung inflammation, fibrosis and immune cell infiltrates in a mouse of the bleomycin model of SSc. **A**, **C** Lung sections of sham treated (Control) or BLM treated (bleomycin model of SSc) WT or *Capns1-ko* mice were stained with H&E (**A**), anti-CD8 (**C**) and F4/80 (**C**). Scale bars, 50 μm,magnification X200. **B**, **D**, **E** An inflammatory score was determined based on histopathological analysis as described in materials and methods (B), or CD8 T cells (**D**) or F4/80 macrophages (**E**) were quantitated using image analysis software. In the WT mice, there was the infiltration of inflammatory cells, widened alveolar septa and pulmonary interstitial fibrosis in lung tissues were found in bleomycin model of SSc group, but no infiltration of inflammatory cells in lung tissues of sham group **A**. Also, there was no infiltration of inflammatory cells in lung tissues of *Capns1-ko* mice in bleomycin model of SSc. In bleomycin model of SSc model, the inflammatory score in lung tissues was significantly higher **B**. Lung tissues containing yellow granulation in the endochylema or nucleus were considered as positive **C**. The number of CD8 positive cells increased in the WT mice of the bleomycin model of SSc group in comparison to WT mice of sham group, and decreased in the *Capns1-ko* mice of the bleomycin model of SSc group with significant difference **D**. The number of F4/80 positive cells increased in the WT mice of the bleomycin model of SSc group in comparison to WT mice of sham group, and decreased in the *Capns1-ko* mice of the bleomycin model of SSc group with significant difference **E**. The number of α-SMA positive cells was similar in the WT mice of the bleomycin model of SSc group in comparison to WT mice of sham group, also similar in the Capns1-ko mice of the bleomycin model of SSc group with no significant difference **F**. The test of Homogeneity of variance showed *P* < 0.1, nonparametric test was applied. Data are mean ± SD. *n* = 8-10, *: *P* < 0.05, *vs.* sham group in WT mice; #: *P* < 0.05, *vs.* bleomycin model of SSc group in WT mice. *OD* optical density.

To explore the role of calpain in ILD in bleomycin model of SSc, we analyzed patho-histological changes in lung tissues in the mouse of the bleomycin model of SSc. H&E staining revealed infiltrations of inflammatory cells, thickened alveolar septa and narrowed alveolar spaces, fibrous thickening of alveolar or bronchiolar walls, widened alveolar septa, and severe distortion of lung structure in lung tissues from bleomycin model of SSc but not sham treated mice, indicative of ILD occurrence in bleomycin model of SSc mice (Fig. 3A). This was confirmed by a significant increase in the inflammation scores in bleomycin model of SSc compared with mouse lung tissues from sham treated mice (*p* < 0.001, Fig. 3B). The infiltration of inflammatory cells was further demonstrated by immunohistochemical analyses for CD8^+^ T cells and F4/80^+^ macrophages and the fibroblast marker of α-SMA (Fig. 3C), with more CD8^+^ T cells (Fig. 3D, *p* = 0.009) and macrophage (Fig. 3E, *p* = 0.004) infiltrations in bleomycin model of SSc compared with mouse lung tissues from sham treated mice. All these patho-histological changes in bleomycin model of SSc mouse lungs were significantly attenuated by deletion of *Capns1* selectively in myeloid cells. But the fibroblast marker of α-SMA was similar in bleomycin model of SSc compared with mouse lung tissues from sham treated mice (Fig. 3F, *p* = 0.0679).

**Fig. 4:**
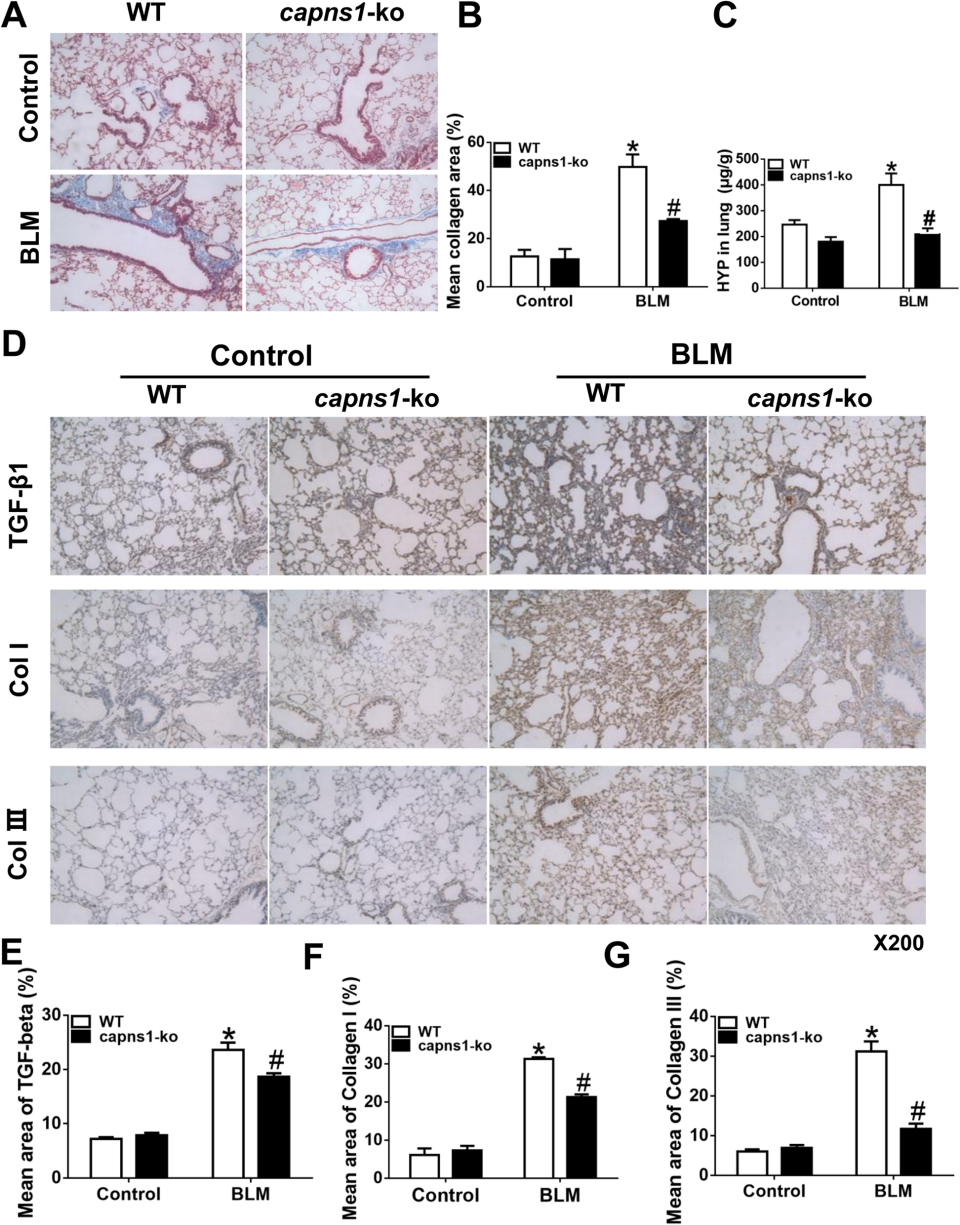
Analyses of fibrosis in lung tissues in a mouse of the bleomycin model of SSc. **A** Lung sections of sham treated (Control) or BLM treated (bleomycin model of SSc) WT or *Capns1-ko* mice were stained with H&E and Masson’s trichrome. Scale bars, 50 μm, magnification X200. **B** Quantitation of mean collagen staining area of lung sections as shown in **A**. **C** The hydroxyproline (HYP) content in lung tissues was determined biochemically and expressed as µg HYP per gram of wet tissue. Masson’s trichrome staining showed a dense deposition of collagen in the pulmonary interstitial tissues in WT mice of the bleomycin model of SSc model, and it significantly increased when the sclerosis was developed in WT mice of the bleomycin model of SSc model **A**. The collagen area rate in lung tissues in sham group was significantly less than that in the WT mice of the bleomycin model of SSc group **B**. The HYP content in lung tissues increased in the bleomycin model of SSc group in comparison to sham group with significant difference in WT mice **C**. In *Capns1-ko* mice of the bleomycin model of SSc model, the collagen area rate and HYP content in lung tissues were increased significantly (**B**, **C**). Data are mean ± SD. *n* = 8. Also, immunohistochemical staining analyses of TGF-β1, collagen I and collagen III in lung tissues in WT and *Capns1-ko* mice of the bleomycin model of SSc were done. **D** Lung sections of sham treated (Control) or BLM treated (bleomycin model of SSc) WT or *Capns1-ko* mice were stained for TGF-β1, Collagen I or Collagen III Scale bars, 50 μm, magnification X200. **E**, **F**, **G** Mean staining areas were quantified for TGF-β1 (**E**), Collagen I (**F**) or Collagen III (**G**). The test of Homogeneity of variance showed *P* < 0.1, nonparametric test was applied. Data are mean ± SD. *n* = 10, *: *P* < 0.05, *vs.* sham group in WT mice; #: *P* < 0.05, *vs.* bleomycin model of SSc group in WT mice. *OD* optical density

To further demonstrate the role of myeloid cell calpain in development of ILD, we analyzed fibrosis in lung tissues. As compared to sham treated mice, bleomycin model of SSc wild-type mice displayed much greater content of collagens in lung tissues (Fig. 4A); however, the amount of collagens was much less in bleomycin model of SSc *Capns1-ko* mice than bleomycin model of SSc wild type mice mouse lung tissues as assessed either by Masson trichrome staining of sections (Fig. 4B) or HYP quantitation of lung tissue (Fig. 4C).

As additional evidence, Lung tissues containing yellow granulation in the endochylema or nucleus were considered as positive (Fig. 4D). The content of TGF-β1 expression increased in the WT mice of the bleomycin model of SSc group in comparison to WT mice of sham group, and decreased in the *Capns1-ko* mice of the bleomycin model of SSc group with significant difference (Fig. 4E). The content of collagen I increased in the WT mice of the bleomycin model of SSc group in comparison to WT mice of sham group, and decreased in the *Capns1-ko* mice of the bleomycin model of SSc group with significant difference (Fig. 4F). Also the content of collagen III increased in the WT mice of SSc group in comparison to WT mice of sham group, and decreased in the *Capns1-ko* mice of SSc group with significant difference (Fig. 4G). BLM-induced SSc was associated with significant increases in TGF-β1, Collagen I and Collagen Ш proteins in wild-type mouse lungs, which were significantly attenuated by C*apns1* knockout (Fig. 4D-G). Taken together, these results indicated that disruption of calpain in myeloid cells reduces ILD in this mouse of the bleomycin model of SSc.

**Fig. 5:**
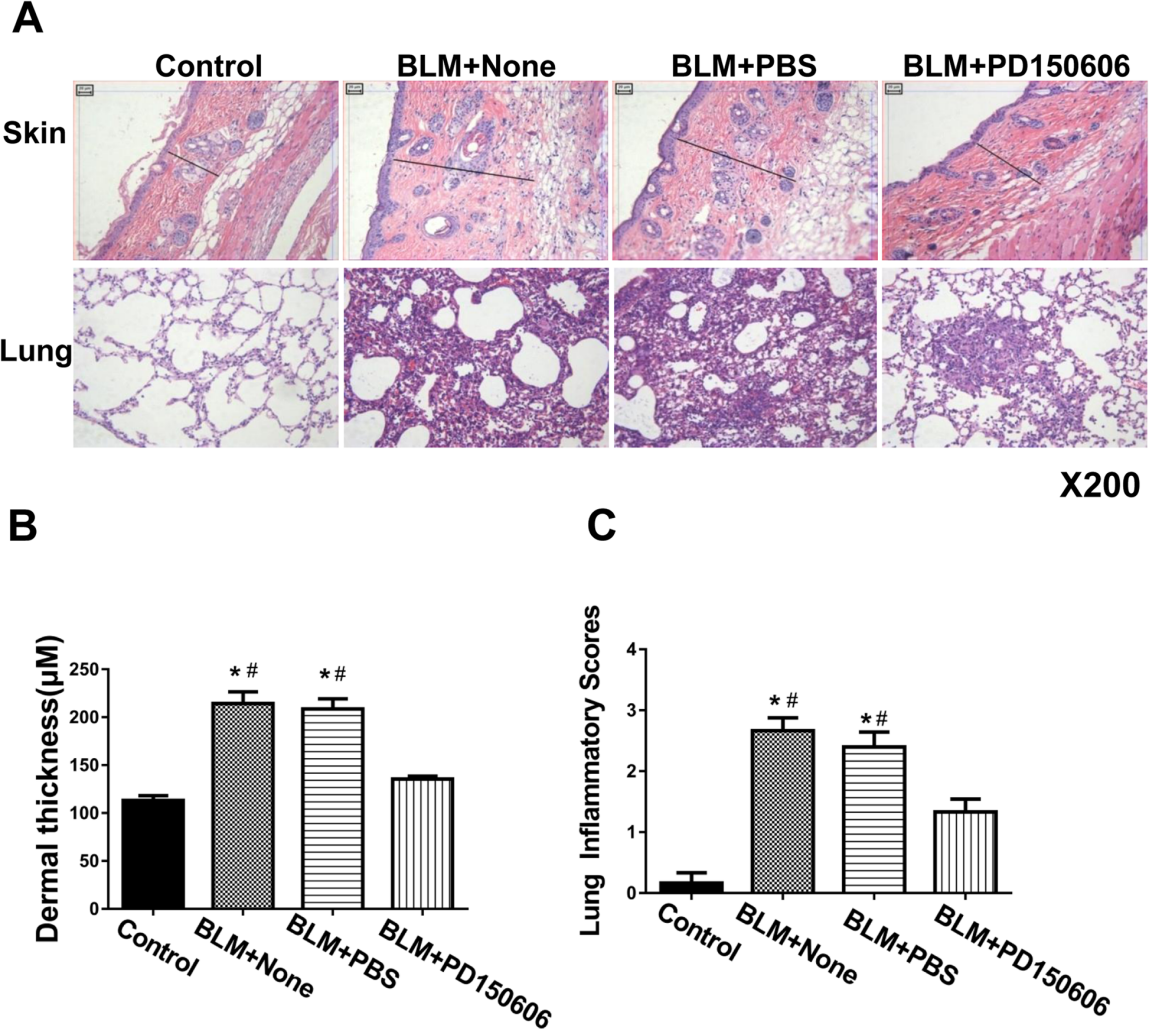
Calpain inhibitor treatment in mouse of the bleomycin model of SSc. **A** C3H/He mice were subjected to either sham treatment (Control) or BLM (bleomycin model of SSc) and then treated with calpain inhibitor (PD150606; 3 mg/kg/day, i.p.) or PBS (vehicle) for 30 days. Skin and lung tissue sections where then assessed by H&E staining. Scale bars, 50 μm, magnification X200. **B** Skin thickness was quantified and expressed in µM. **C** Lung inflammatory scores were determined based on histopathological analysis as described in materials and methods. Data are mean ± SD. The test of Homogeneity of variance showed *P* > 0.1, AVONA test was applied. *n* = 7, * *P* < 0.05 in comparison to Sham group; ^#^ *P* < 0.05 in comparison to BLM + PD150606 group

To investigate whether pharmacological inhibition of calpain has protective effects in SSc mice similar to deletion of *Capns1*, we injected bleomycin model of SSc mice with PD150606, a selective calpain inhibitor. In bleomycin model of SSc group, the thickened dermis with cellular infiltrates was shown (Fig. 5A). In the BLM + None and BLM + PBS mice, there was the infiltration of inflammatory cells, widened alveolar septa and pulmonary interstitial fibrosis in lung tissues were found in bleomycin model of SSc group, but no infiltration of inflammatory cells in lung tissues of sham group (Fig. 5A). Also, there was few infiltration of inflammatory cells in lung tissues of BLM + PD150606 group (Fig. 5A). In bleomycin model of SSc, the thickness of skin was significantly higher (Fig. 5B). The inflammatory score of lung tissues was significantly higher in BLM + None and BLM + PBS mice (Fig. 5C).Administration of PD150606 did not induce any adverse effects on skin or lung in sham treated mice, but it significantly reduced skin thickening and it attenuated ILD in bleomycin model of SSc mice as determined by patho-histological analysis (Fig. 5A-C). These results provide evidence implicating calpain expression in myeloid cells in ILD in this mouse of the bleomycin model of SSc.

### Disruption of calpain prevents macrophage polarization toward an M1 phenotype in lung tissues of the bleomycin model of SSc mice

**Fig. 6:**
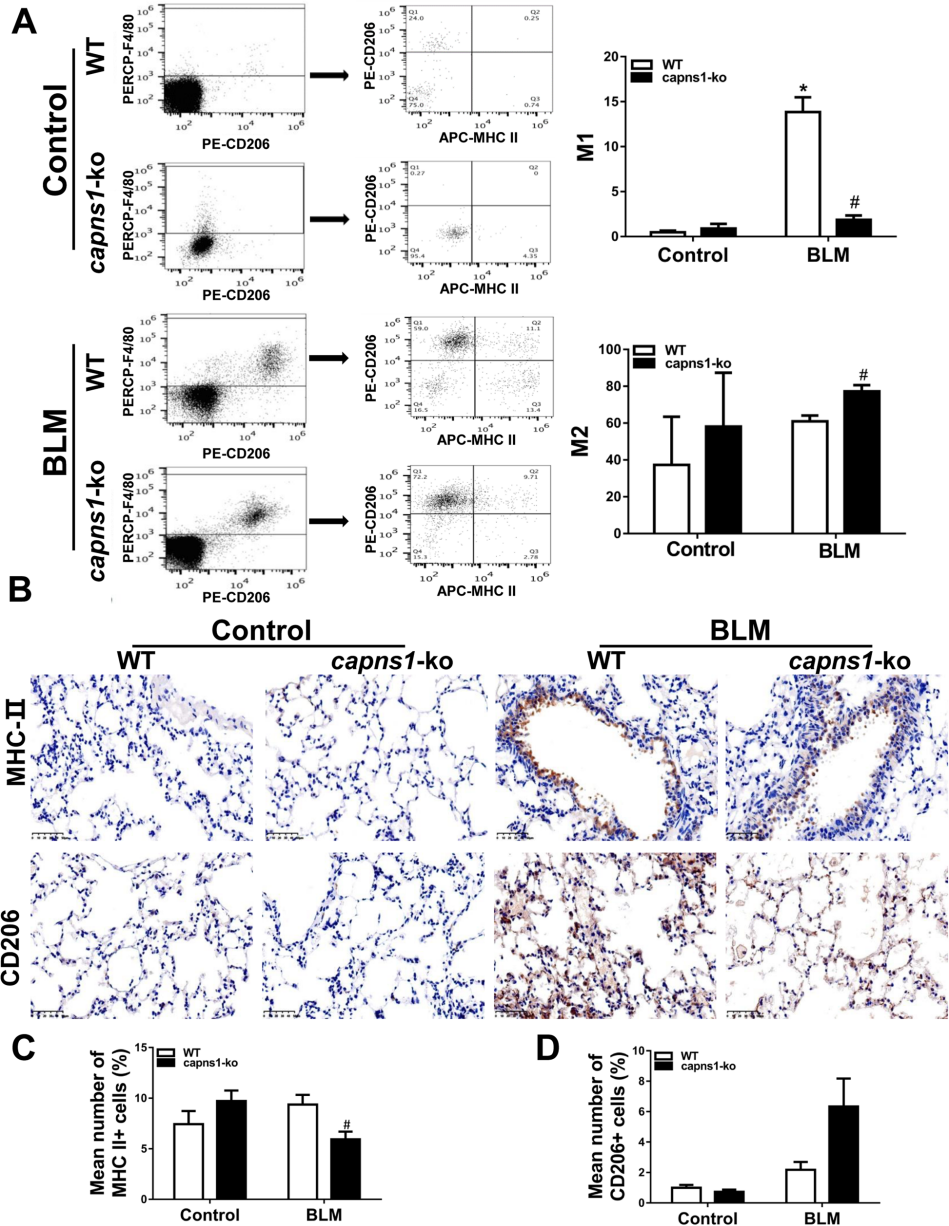
Flow cytometry analysis of M1 and M2 macrophages in lung tissues of the bleomycin model of SSc mice. **A** Single cell suspensions of lung tissues from sham treated (Control) or BLM treated (bleomycin model of SSc) WT or *Capns1-ko* mice were subjected to flow cytometry. F4/80^+^ macrophages were assessed for expression of MHCII and CD206 as markers of M1 and M2 macrophages, respectively. The number of M1 macrophages was significantly higher in WT mice of the bleomycin model of SSc model and decreased in *Capns1-ko* mice in the bleomycin model of SSc. The number of M2 macrophages was not significantly higher in WT mice of the bleomycin model of SSc and increased significantly in *Capns1-ko* mice in the bleomycin model of SSc(A). Also, it was further demonstrated by immunohistochemical analyses for CD206 and MHC-II **B**. Scale bars, 50 μm.The MHC-II^+^cells were decreased in bleomycin model of SSc compared with the sham treated mice **C**, but the CD206^+^cells was similar between bleomycin model of SSc and sham treated mice **D**. The test of Homogeneity of variance showed *P* < 0.1, nonparametric test was applied. Data are mean ± SD. *n* = 8, *: *P* < 0.05, *vs.* sham treated WT mice; #: *P* < 0.05, *vs.* bleomycin model of SSc WT mice. *OD* optical density

**Fig. 7:**
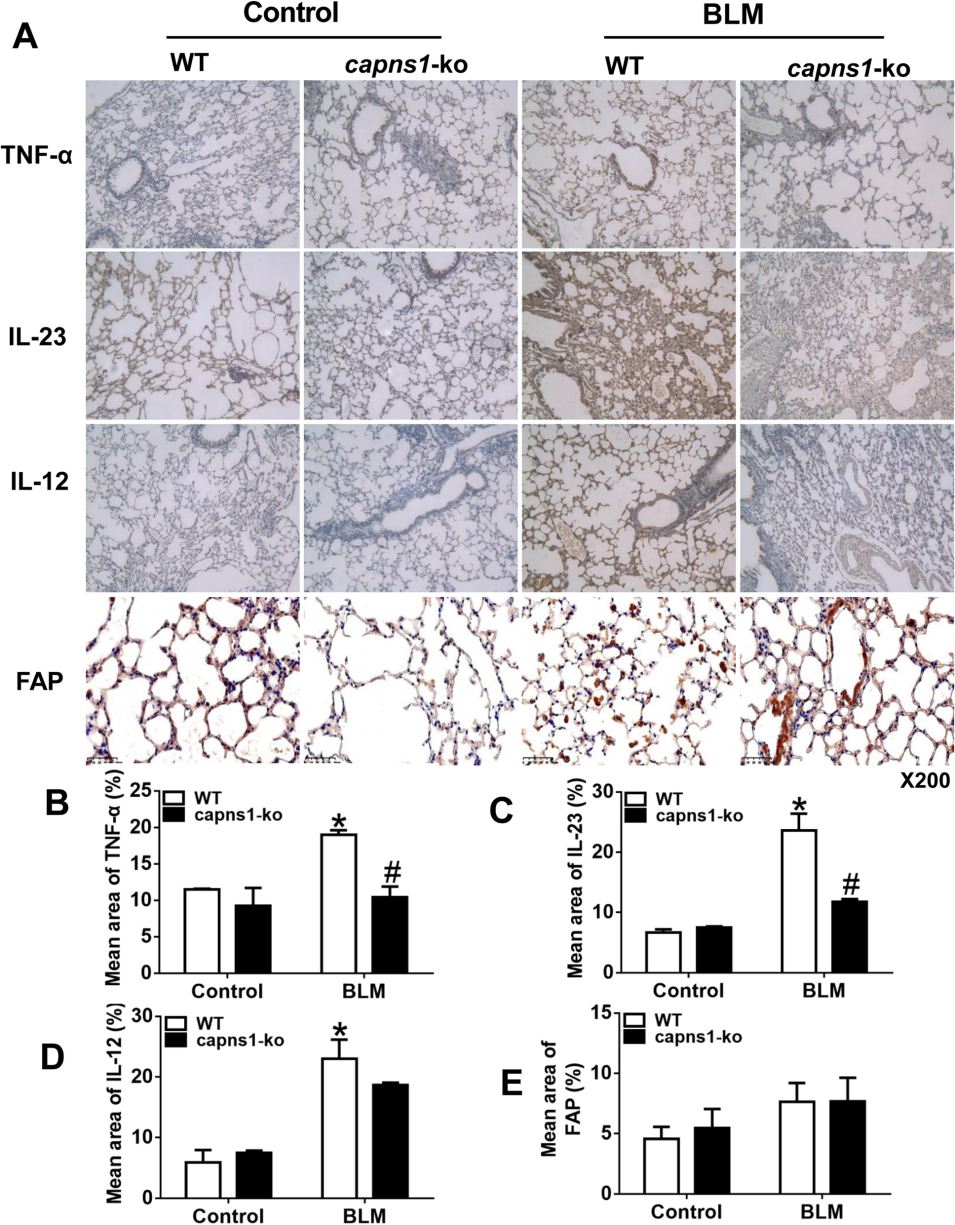
Immunohistochemical analysis of TNFα, IL-12 and IL-23 in lung tissues of the bleomycin model of SSc mice. **A** Lung sections of sham treated (Control) or BLM treated (bleomycin model of SSc) WT or *Capns1-ko* mice were stained for TNFα, IL-23, IL-12 or FAP. Scale bars, 50 μm, magnification X200. (B, C, D,E) Mean staining area were quantified for TNFα **B**, IL-23 **C**, IL-12 **D** or FAP. **E**. The test of Homogeneity of variance showed *P* < 0.1, nonparametric test was applied. Data are mean ± SD. *n* = 10, *: *P* < 0.05, *vs.* sham treated WT mice; #: *P* < 0.05, *vs.* bleomycin model of SSc WT mice. *OD*: optical density

Having shown that infiltrations of macrophages and inflammation were increased in lung tissues of the bleomycin model of SSc mice and that both were attenuated by deletion of *Capns1*, we hypothesized that calpain might play a role in the polarization of macrophages to an M1 phenotype in the development of ILD. To address this, we analyzed the frequency of F4/80^+^MHC II^+^ and F4/80^+^CD206^+^ cells in the lungs of the bleomycin model of SSc and sham treated mice. F4/80 is a marker of active macrophages and MHC II is high in M1 macrophages while CD206 is high in M2 macrophages [29]. Flow cytometry analysis revealed that the percentage of F4/80^+^MHC II^+^ M1 cells was significantly higher in bleomycin model of SSc compared with sham treated mice (13.83 ± 2.88 vs. 0.47 ± 0.32, *p* = 0.001, Fig. 6A), and this increase in percentage of F4/80^+^MHC II^+^ M1 cells was attenuated in bleomycin model of SSc *Capns1-ko* mice (13.83 ± 2.88 vs. 1.86 ± 0.83, *p* = 0.002, Fig. 6A). Although the percentage of F4/80^+^ CD206^+^ M2 cells was similar between bleomycin model of SSc and sham treated mice (60.93 ± 5.46 vs. 37.27 ± 45.37, *p* > 0.05, Fig. 6A), it was slightly higher in bleomycin model of SSc *Capns1-ko* mice. Also, it was further demonstrated by immunohistochemical analyses for CD206 and MHC- II (Fig. 6B). The MHC-II^+^cells were decreased in bleomycin model of SSc compared with the sham treated mice(*p* = 0.0474, Fig. 6C), but the CD206^+^cells was similar between bleomycin model of SSc and sham treated mice (*p* = 0.1208, Fig. 6D). In support of pro-inflammatory phenotype of M1 macrophages, bleomycin model of SSc mouse lungs displayed a significant increase in M1 macrophage-expressed cytokines including TNF-α, IL12 and IL23, which was not observed in bleomycin model of SSc *Capns1-ko* mice (Fig. 7). Lung tissues containing yellow granulation in the endochylema or nucleus were considered as positive(Fig. 7A). The content of TNF-α expression increased in the WT mice of the bleomycin model of SSc group in comparison to WT mice of sham group, and decreased in the *Capns1-ko* mice of the bleomycin model of SSc group with significant difference (Fig. 7B). The content of IL-23 increased in the WT mice of the bleomycin model of SSc group in comparison to WT mice of sham group, and decreased in the *Capns1-ko* mice of the bleomycin model of SSc group with significant difference (Fig. 7C). The content of IL-12 increased in the WT mice of the bleomycin model of SSc group in comparison to WT mice of sham group, but did not decrease significantly in the *Capns1-ko* mice of the bleomycin model of SSc group with significant difference (Fig. 7D). The content of FAP(fibroblast activation protein) was similar in the WT mice of the bleomycin model of SSc group in comparison to WT mice of sham group (Fig. 7E).

These results suggest that calpain may promote macrophage polarization toward M1 phenotype.

### *Inhibition of calpain increases PI3K*/*AKT1 signaling in lung tissues of the bleomycin model of SSc mice*

**Fig. 8:**
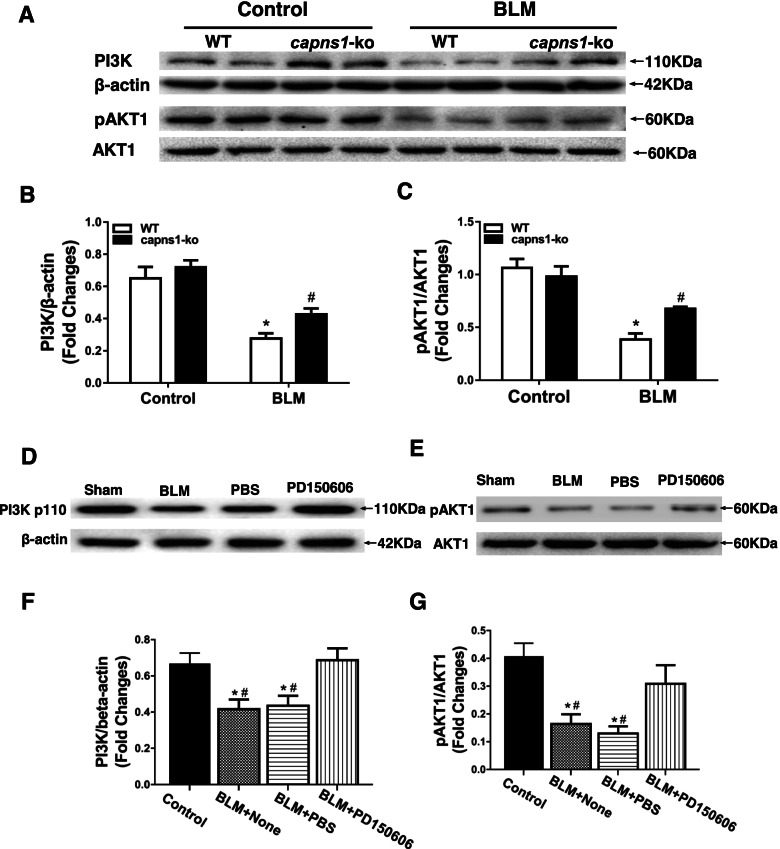
Analysis of PI3K levels and activated AKT1 in lung tissues of the bleomycin model of SSc mice. (A)Lung tissues of sham treated (Control) or BLM treated (bleomycin model of SSc) WT or *Capns1-ko* mice were subjected to SDS PAGE and immunoblotting for PI3K, AKT1, pAKT1 and β-actin. (B, C) Levels of the proteins assessed in (A) were quantified by densitometry. (D, E) C3H/He mice were subjected to either sham treatment (Control) or BLM and then treated with PBS or calpain inhibitor (PD150606; 3 mg/kg/day, i.p.) or PBS (vehicle) for 30 days. Lung tissues were then analyzed as in (A); and (F, G) quantified as in (B, C). The PI3K and pAKT1 protein in different groups were done by Western blotting analysis (A). The test of Homogeneity of variance showed *P* < 0.1, nonparametric test was applied. The grayscale levels of PI3K and pAKT1were much greater in the WT mice of SSc model versus WT mice in sham group with significant difference and significantly decreased in *Capns1-ko* mice in SSc model in comparison to WT mice in SSc model (B,C). Also, the PI3K and pAKT1 protein in different groups were done by Western blotting analysis (D). The test of Homogeneity of variance showed *P* > 0.1, AVONA test was applied. The grayscale level of PI3K was much greater in the BLM + None and BLM + PBS group versus sham group with significant difference and significantly increased in BLM + PD150606 group in comparison to BLM + None group (F).The pAKT1 and AKT1 protein in different groups were done by Western blotting analysis (E). Also, the grayscale level of pAKT1 was much greater in the BLM + None and BLM + PBS group, esp. in BLM + PBS group, versus sham group with significant difference and significantly increased in BLM + PD150606 group in comparison to BLM + None group (G). Data are mean ± SD. *n* = 7, * *P* < 0.05 in comparison to Sham group; ^#^ *P* < 0.05 in comparison to BLM + PD150606 group

To explore potential molecular mechanisms by which calpain contributed to ILD in bleomycin model of SSc mice, we measured the protein levels of PI3K and phosphorylated AKT1 in lung tissues. When compared with sham treated mouse lungs, the protein levels of both PI3K and phosphorylated AKT1 were significantly reduced in bleomycin model of SSc mouse lungs (Fig. 8). However, the levels of PI3K (0.72 ± 0.08 vs. 0.43 ± 0.06, *p* = 0.034, Fig. 8A and 8B) and phosphorylated AKT1 (0.98 ± 0.17 vs. 0.68 ± 0.03, *p* = 0.002, Fig. 8A and 8C) were relatively higher in bleomycin model of SSc *Capns1-ko* compared with bleomycin model of SSc wild-type lung tissues. Consistently, the protein levels of PI3K (0.6863 ± 0.06622 vs. 0.4163 ± 0.05752, *p* = 0.0053, Fig. 8D and 8F) and phosphorylated AKT1 (0.3085 ± 0.06719 vs. 0.164 ± 0.03512, *p* = 0.0299, Fig. 8E and 8G) were also higher in lung tissues from PD150606-treated compared with vehicle bleomycin model of SSc wild-type mice. These results suggest that inhibition of calpain increases PI3K/AKT1 signaling in lungs of the bleomycin model of SSc mice.

## Discussion

Calpain has been implicated in pulmonary arterial hypertension [12]. Also, our lab recently reported an increase in serum calpain activity, which correlated with elevation of HMGB1 in patients with SSc or SSc-ILD [30]. The serum calpain activity and HMGB1 levels may serve as measures of ILD in patients with SSc [30]. However, a mechanistic role of calpain in bleomycin model of SSc-ILD has never been reported. The major findings of this study are as follows: (1) Deletion of *Capns1* in myeloid cells alleviated both lung and skin inflammation and fibrosis in bleomycin model of SSc; (2) Bleomycin model of SSc induced a significant increase in TGF-β1, Collagen I and Collagen Ш proteins in wild-type mouse lungs, which were significantly attenuated by *Capns1-ko*; and (3) The protective effects of *Capns1-ko* were recapitulated by systemic pharmacological inhibition of calpain in bleomycin model of SSc-related ILD and were associated with prevention of lung macrophage polarization toward the M1 phenotype, and decreased productions of pro-inflammatory cytokines including TNF-α, IL-12 and IL-23 in bleomycin model of SSc mouse lung tissues. To the best of our knowledge, our study demonstrates for the first time that myeloid cell calpain plays an important role in bleomycin model of SSc-related ILD.

Calpain has been implicated in vascular remodeling and collagen synthesis [31, 32]. Several studies have reported that inhibition of calpain attenuates bleomycin-induced pulmonary fibrosis in mice [11, 33–35]. Furthermore, a recent study has demonstrated that calpain activation by the renin-angiotensin system induces collagen-I synthesis and pleural fibrosis [33]. This study extends the role of calpain in lung fibrosis to bleomycin model of SSc-related ILD. Several lines of evidence support this conclusion. First, inhibition of calpain attenuated pathological fibrotic changes in lungs of the bleomycin model of SSc mice. Second, inhibition of calpain reduced the contents of hydroxyproline (HYP) in lungs of the bleomycin model of SSc mice. Third, the increased expression of TGF-β1, collagen I and collagen in bleomycin model of SSc-ILD was attenuated by *Capns1*knockout. Additionally, both *Capns1*-ko and pharmacologic inhibition of calpain reduced inflammation in lung tissues of the bleomycin model of SSc-ILD mice. Future study is warranted to support the role of calpain by analyzing lung tissue samples in patients with bleomycin model of SSc-ILD.

Macrophages are crucial players in orchestrating inflammation and fibrosis in bleomycin model of SSc [36]. Macrophages can be activated by a variety of stimuli and polarized to functionally different phenotypes. Two distinct subsets of macrophages have been proposed, including classically activated (M1) and alternatively activated (M2) macrophages. M1 macrophages express a series of proinflammatory cytokines, chemokines and effector molecules, such as IL-12, IL-23, TNF-α, iNOS and MHCI/II. In contrast, M2 macrophages express a wide array of anti-inflammatory molecules, such as IL-10, TGF-β, and arginase1 in addition to the mannose receptor CD206 [37]. An important finding of the present study is that loss of calpain in macrophages prevents macrophage polarization toward the M1 phenotype in lung tissues of *Capns1*-ko SSc-ILD compared with wild-type mice, underscoring a critical role of calpain in macrophage polarization toward M1 proinflammatory phenotype. This was supported by our recent report [20]. Given the role of macrophages in inflammation and fibrosis, two features of the bleomycin model of SSc-ILD, our findings suggest the following model: calpain activation promotes macrophage M1 polarization thereby mediating inflammation and fibrosis, leading to ILD in bleomycin model of SSc mice.

The mechanisms by which calpain promotes macrophage polarization toward the M1 phenotype remain unknown. PI3K/AKT1 signaling as calpain has been reported to modulate both PI3K and AKT1 in inflammation of lung tissues in different models of pulmonary artery and asthma [38, 39]. But, in several researches it has been reported that calpain cleaves PI3K proteins in vitro resulting in a reduction of PI3K lipid kinase activity [40], and this regulates endogenous PI3K protein levels in vivo [41]. Thus, calpains have an inhibitory role in regulation of the PI3K/AKT pathway activity [41]. In line with these previous reports, the present study showed that both knock-out of *Capns1* and pharmacological inhibition of calpain increased the protein levels of PI3K and AKT activation in lung tissues of the bleomycin model of SSc-ILD mice. It is therefore possible that inhibition of the PI3K/AKT pathway may mediate the role of calpain in macrophage polarization toward the M1 phenotype in bleomycin model of SSc-ILD as the PI3K/AKT signaling axis has been implicated in association with macrophage polarization.

It is important to emphasize that deletion of myeloid cell *Capns1* also reduced dermal sclerosis in bleomycin model of SSc mice. Future study is needed to determine how calpain is implicated in dermal sclerosis and whether calpain could be a therapeutic target for dermal sclerosis. Additionally, currently we do not know whether attenuated dermal sclerosis resulting from the deletion of myeloid cell *Capns1* contributes to the development of the bleomycin model of SSc-ILD.

## Conclusions

In conclusion, this is the first study to demonstrate that the role of myeloid cell calpain a potential therapeutic target for bleomycin model of SSc-related ILD. We have provided evidence demonstrating that myeloid-specific calpain is important in the development of the bleomycin model of SSc-ILD. This role of calpain is associated with macrophage polarization towards to the M1 type and subsequent inflammation and fibrosis, furthermore, mice treated with a calpain inhibitor reduced the inflammation and fibrosis in bleomycin model of SSc-ILD presumably through association with PI3K/AKT signaling. Thus, calpain may be a potential therapeutic target for bleomycin model of SSc-ILD.

## Data Availability

The datasets generated and/or analyzed in this study are available from the corresponding author upon reasonable request.
